# Single-cell-based evaluation of sperm progressive motility via fluorescent assessment of mitochondria membrane potential

**DOI:** 10.1038/s41598-017-18123-1

**Published:** 2017-12-20

**Authors:** Natalina Moscatelli, Barbara Spagnolo, Marco Pisanello, Enrico Domenico Lemma, Massimo De Vittorio, Vincenzo Zara, Ferruccio Pisanello, Alessandra Ferramosca

**Affiliations:** 1 Center for Biomolecular Nanotechnologies @UNILE, Istituto Italiano di Tecnologia, Via Barsanti, I-73010 Arnesano (LE), Italy; 2 Dipartimento di Scienze e Tecnologie Biologiche ed Ambientali, Università del Salento, Via Provinciale Lecce-Monteroni, I-73100 Lecce, Italy; 3 Dipartimento di Ingegneria Dell’Innovazione, Università del Salento, Via Provinciale Lecce-Monteroni, I-73100 Lecce, Italy

## Abstract

Sperm cells progressive motility is the most important parameter involved in the fertilization process. Sperm middle piece contains mitochondria, which play a critical role in energy production and whose proper operation ensures the reproductive success. Notably, sperm progressive motility is strictly related to mitochondrial membrane potential (MMP) and consequently to mitochondrial functionality. Although previous studies presented an evaluation of mitochondrial function through MMP assessment in entire sperm cells samples, a quantitative approach at single-cell level could provide more insights in the analysis of semen quality. Here we combine laser scanning confocal microscopy and functional fluorescent staining of mitochondrial membrane to assess MMP distribution among isolated spermatozoa. We found that the sperm fluorescence value increases as a function of growing progressive motility and that such fluorescence is influenced by MMP disruptors, potentially allowing for the discrimination of different quality classes of sperm cells in heterogeneous populations.

## Introduction

Human spermatozoa rely mainly on two metabolic pathways to obtain energy: anaerobic glycolysis and mitochondrial oxidative phosphorylation (OXPHOS)^1,2^. Glycolysis takes place mainly in fibrous sheath of the flagellum, whereas OXPHOS occurs in mitochondria, which are localized exclusively in the sperm mid-piece. Sperm mitochondria are unique organelles that participate in many crucial processes, the major being the synthesis of adenosine triphosphate (ATP) through the sophisticated mechanism of the oxidative phosphorylation (OXPHOS)^1,2^. This process requires the coordinated operation of two main components: the respiratory chain and the ATP-synthase, both located in the inner mitochondrial membrane. The mitochondrial respiratory chain is involved in the transport of reducing equivalents from some electron-donors to the molecule of oxygen with the final formation of water. The energy released from these oxidation/reduction reactions is used to drive the synthesis of ATP from adenosine diphosphate (ADP). A strict coupling is therefore required between respiration, which is the electron transfer through respiratory chain complexes, and phosphorylation, which is necessary to synthesize ATP. In sperm cells, OXPHOS occurs at the level of the middle piece, where mitochondria are located, and it provides the energy necessary to sustain flagellar movements^1^.

Several studies reported that mitochondrial functionality is associated with sperm quality, especially with sperm motility, an essential factor in the diagnosis of male infertility. According to this hypothesis, structural and functional alterations are usually found in mitochondria from asthenozoospermic subjects^1^ (e.g. with progressive motility <32% according to WHO criteria^3^). However, not only motility but also several essential sperm functions require ATP as an energy source^1,4^. In particular, low activity of sperm mitochondrial enzymes, including electron transfer chain complexes, also correlates with sperm parameters, such as decreased concentration, vitality and motility^5^. The importance of mitochondrial functionality was also extended to hyperactivated motility and to the strictly associated phenomenon of sperm capacitation^6^, which is characterized by an increase in flagellar bend amplitude and, usually, beat asymmetry. Therefore, a careful and detailed investigation of mitochondrial functionality of spermatozoa could provide more insights on the role of these organelles in the overall quality of the gametes.

Various methodological approaches have been developed to assess mitochondrial functions, such as spectrophotometric assays^5,7^, polarographic methods^8–10^ or cytofluorimetry^11–20^. In particular, spectrophotometry is used to measure the activity of specific enzymes involved in mitochondrial metabolism. Polarography measures the rate of change in oxygen concentration in solution and, since oxygen is the final electron acceptor of the respiratory chain, it provides a direct measurement of mitochondrial activity. Flow cytometry, instead, can be used to estimate membrane potential across the mitochondrial inner membrane, which is an indirect method of assessing mitochondrial respiration activity.

In clinical studies, mitochondrial function is generally monitored using cationic fluorescent probes, such as MitoTracker or JC-1^21^. As shown in^21^, these types of luminescent molecules can be used to selectively stain mitochondria in the sperm cell middle piece depending on the mitochondria membrane potential (MMP), and they can be used to discriminate cells with active and non-active mitochondria. A step further was done in 2011 by Paoli and coworkers^22^ by using JC-1 in a cytofluorymetry-based evaluation of sperm. With respect to MitoTracker, JC-1 has the peculiarity of creating agglomeration that red-shifts the emission wavelength. With this approach, Paoli *et al*. were able to demonstrate that both human sperm motility and viability are associated with MMP. These observations suggest that MMP can be considered a parameter of particular clinical interest, since it is an indicator that best reflects mitochondrial function and, in turn, sperm functionality. Therefore, the identification of high MMP in human sperm cells is an appropriate test to determine sperm quality and it has a good potential to become a routine analysis to evaluate the fertilizing ability of sperm and for the diagnosis of male infertility^23^. Although the link between high MMP and high viability and motility is clear from the literature, to the best of our knowledge currently available approaches only distinguish between the existence or not of a detectable fluorescence signal from the analyzed cells.

Here we exploit laser scanning confocal microscopy and MitoTracker staining to assess the fluorescence intensity distribution among the sample at the single-cell level, since MitoTracker-positive sperm cells constitute the better quality and more functional gametes subpopulation^20^.

We found that the average fluorescence value increases as a function of the progressive motility and oxygen consumption. The obtained data suggest that this approach, based on the distribution of a specific fluorescent probe at the level of the inner mitochondrial membrane, can be exploited not only to distinguish between high and low motility samples, but also to have a quantitative assessment of mitochondria functionality.

## Results

### Identification of MMP levels thorough fluorescent staining of inner mitochondria membrane

**Figure 1:**
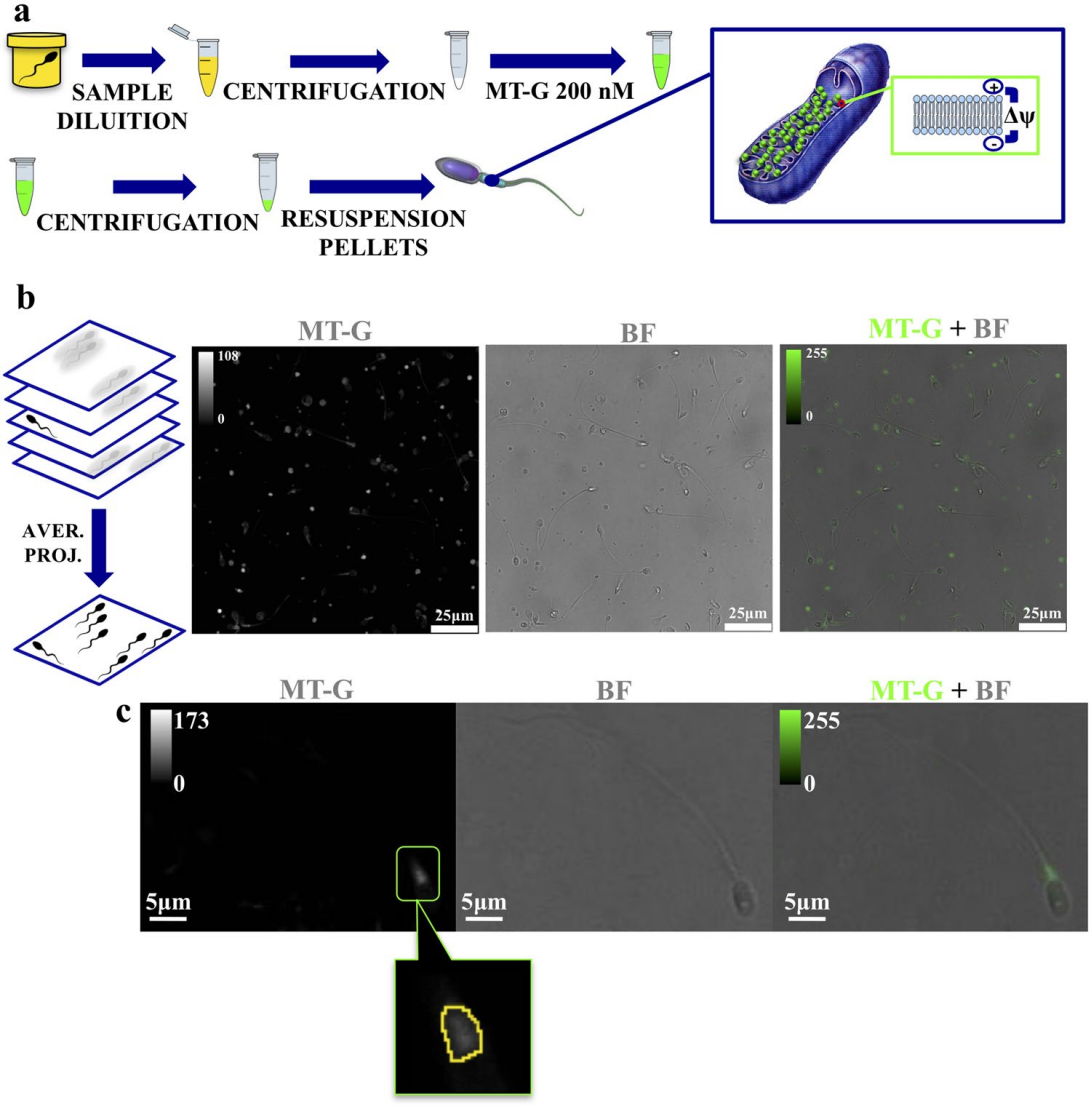
Processing method and sample analysis. Panel (a) Steps of MitoTracker-Green (MT-G) staining. From left to right and from top to bottom: sperm sample dilution, centrifugation at 10000 g for 5 minutes at room temperature, pellet resuspension in 200 nM MitoTracker Green FM solution and incubation for 15 minutes at 37 °C, a further centrifugation and pellets resuspension in saline water (1 ml). The zoom schematically shows that the fluorescent probe is distributed in sperm inner mitochondrial membrane. Δψ indicates membrane potential. Panel (b) From left to right: Schematic representation of the average projection (aver. prog.) process and the so obtained fluorescence (MT-G), bright-field (BF) and overlay (MT-G + BF) images obtained through Laser Scanning Confocal channels. Color scales for fluorescence images are reported in arbitrary units. Panel (c) Fluorescence (MT-G), bright-field (BF) and overlay (MT-G + BF) images of an isolated cell. The bottom image represents a zoom on a sperm-cell middle piece, with the yellow-outlined area highlighting the region used for quantifying μ_PL_ and M_PL_ figures of merit. Color scales for fluorescence images are reported in arbitrary units.

To develop the staining protocol, semen samples from different patients were collected as described in methods section. The staining protocol is summarized in Fig. 1a. After a dilution, the sample was centrifuged to separate cells from the seminal fluid. The obtained pellet was incubated with 200 nM solution of MitoTracker in order to obtain a selective fluorescence from the inner mitochondria membrane in the middle piece^21^. Although MitoTracker has been shown to be a promising fluorescent indicator of high mitochondria membrane potential^23^, to the best of our knowledge its suitability to investigate the membrane potential at the single**-**cell level and to discriminate between different progressive motility levels in a heterogeneous sample, has not been shown yet. To this purpose we used a fluorescence detection method based on a laser scanning confocal (LSC) microscope. After optimizing acquisition parameters (laser excitation wavelength and power, and gain of the acquisition system), we found that the green version of MitoTracker (MT-G) allows to reach optimized acquisition conditions also for low laser excitation power, minimizing the influence of photo-bleaching on the measured signal, in particular if compared with its red shifted version MitoTracker Red. In this latter case, a signal to noise ratio comparable with the one obtained for MT-G was observed at two-time higher excitation power. 200 nM was selected as the best concentration of MT-G to stain only active mitochondria from live sperm cells with the lowest progressive motility, and to distinguish them from the noise. On the other hand, this probe concentration is the maximum recommended by the datasheet guidelines to reduce artefacts, mitochondrial toxicity and to improve the selectivity of this probe, because at higher concentration the probe stains other cell structures^24^.

For LSC analysis the sample was cast into the center of a petri dish and 6 field of view of 100 × 100 µm were randomly chosen in the proximity of the center of the petri, in an overall 1 × 1 cm area. For each field of view, an average projection was obtained starting from a 0.5µm-spaced z-stack acquired for an overall z-spanning of 2 µm (Fig. 1b). Excitation wavelength was set at 490 nm (power of 12%). A typical output of this measurement is shown in Fig. 1b, showing fluorescence from both sperm cells and other cell types present in the seminal fluid. All detected sperm cells in each z-stack show selective staining of the middle piece, as expected. For each sperm cell, both average and maximum photoluminescence (PL) intensity from the entire middle piece (hereafter referred to as μ_PL_ and M_PL_, respectively) were measured (Fig. 1c) for each cell and used as a figure of merit for the statistical analysis described below. Since not all the sperm cells lie on the petri dish bottom surface, the average projection of the z-stack allows to take into account all the cells in the region of interest.

**Figure 2:**
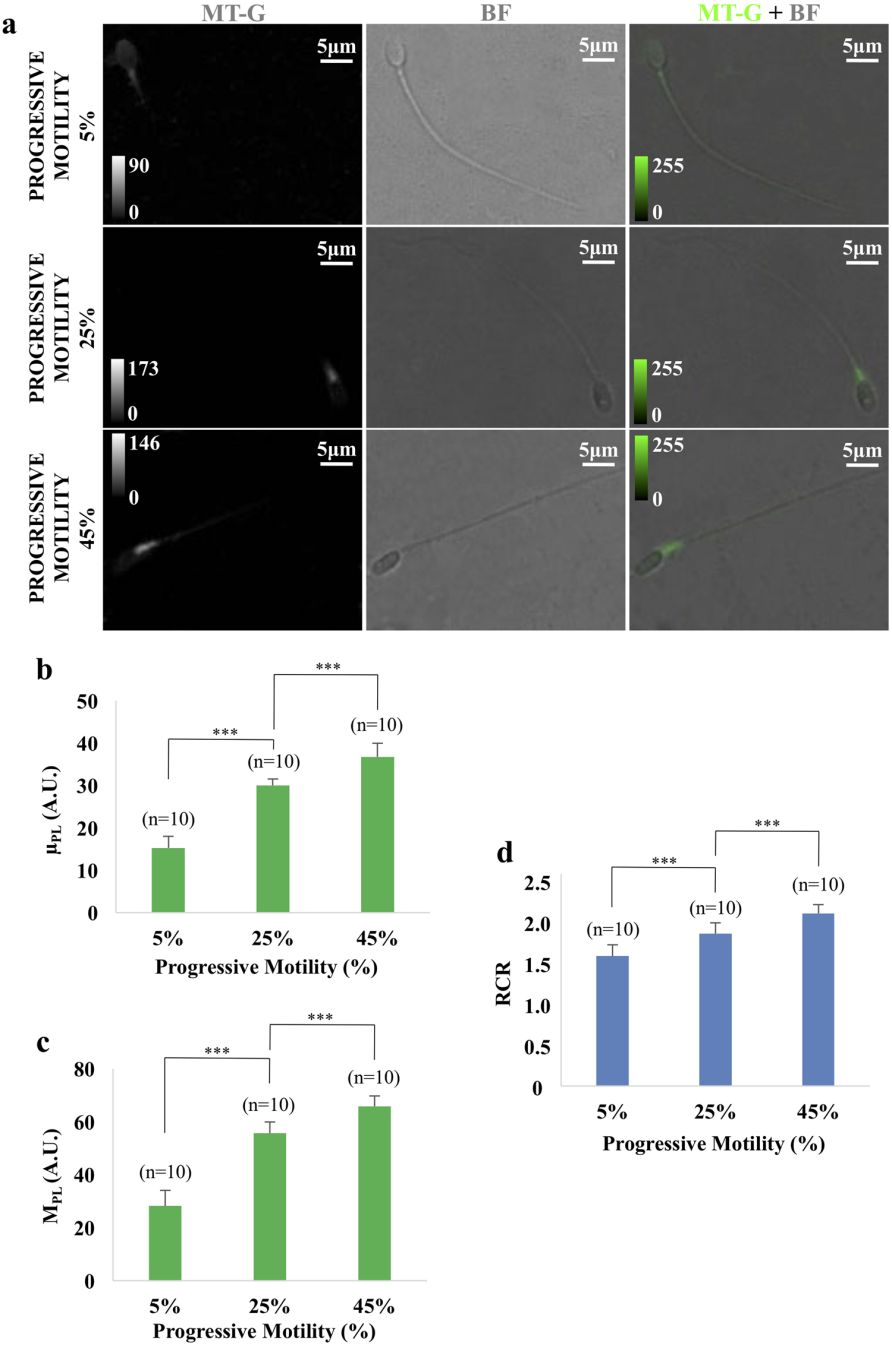
Link between progressive motility and photoluminescence intensity. Panel (a) Representative images of isolated spermatozoa with different progressive motility (i) 5%, (ii) 25% and (iii) 45%, from top to bottom. From left to right fluorescence (MT-G), bright field (BF) and overlay (MT-G + BF) images are displayed. Color scales for fluorescence images are reported in arbitrary units (A.U.). Panel (b) Bar graph of μ_PL_ in the three sample groups (***p < 0.001; p (5% vs. 25%) = 5.20 × 10^−10^; p (25% vs. 45%) = 0.000047). Data are reported as mean value and error bars represent standard deviations on n = 10 samples. μ_PL_ represents the average value of photoluminescence intensity from the entire middle piece. A.U. represents arbitrary units fluorescence intensity. Panel (c) Bar graph of M_PL_ in the three sample groups (***p < 0.001; p (5% vs. 25%) = 1.59 × 10^−9^; p (25% vs. 45%) = 0.000029). Data are reported as mean value and error bars represent standard deviations on n = 10 samples. M_PL_ represents the maximum value of photoluminescence intensity from the entire middle piece. A.U. represents arbitrary units fluorescence intensity. Panel (d) Bar graph of the Respiratory Control Ratio (RCR) average in the three sample groups with growing progressive motility (***p < 0.001; p (5% vs. 25%) = 0,00023; p (25% vs. 45%) = 0,00025). Data are reported as mean value and error bars represent standard deviations on n = 10 samples.

Three human sperm sample groups with growing progressive motility (which measures the ability of sperm cells to move forward on a straight line) were selected: (i) 45 +/− 0.5% (normozoospermic samples, n = 10), (ii) 25 +/− 0.5% (asthenozoospermic samples, n = 10) and (iii) 5 +/− 0.5% (severe asthenozoospermic samples, n = 10). The samples (n = 10 × 3 progressive motility group) were processed and analyzed in different batches on different days. Representative LSC images of isolated sperm cells in three different samples, from each progressive motility group, are shown in Fig. 2a. As a general trend, the mean values of both M_PL_ and μ_PL_ increase as a function of progressive motility. This is summarized in the bar graphs displayed in Fig. 2b and c: sperm cells in the group with the highest progressive motility show the highest mean value for both figures of merit, while average PL decreases in the case of 25% and 5% progressive motility (*p* < 0.001 for all comparisons). This well matches also with Respiratory Control Ratio (RCR) data on same sample groups (Fig. 2d) obtained by the polarographic method, which are a direct measurement of mitochondria functionality through the determination of oxygen consumption^8^.

**Figure 3:**
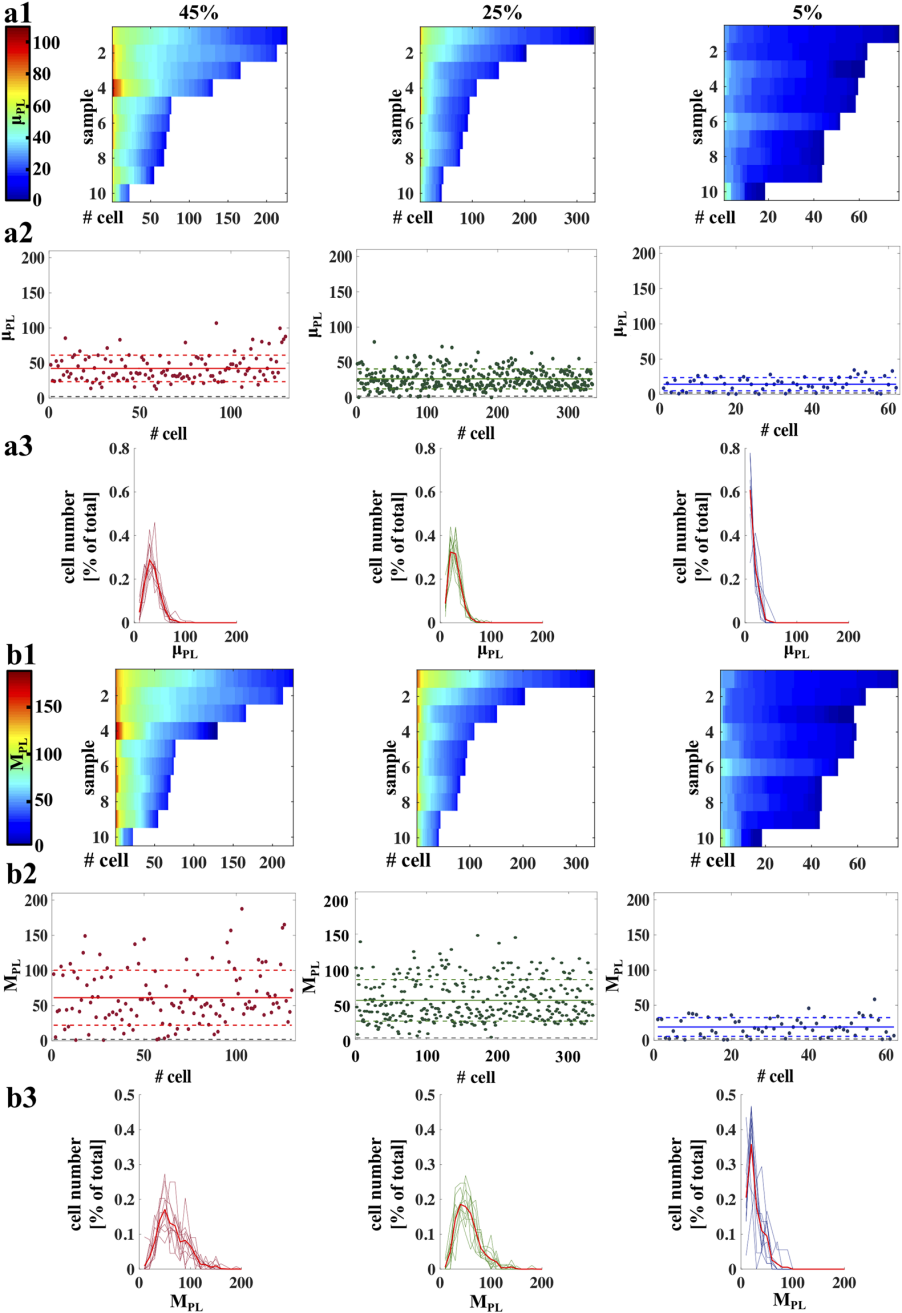
Statistical analysis. Panel (a1) Average value of photoluminescence intensity from the entire middle piece (μ_PL_) intensities from 10 samples for each progressive motility category (reported on the top of each plot). The color bar is reported in arbitrary units (A.U.): from blue to red, the bar shows growing μ_PL_ values. Therefore, each point of the color map represents the μ_PL_ intensity recorded from a single cell in one of the 10 samples. Data are sorted by decreasing cell number and fluorescence intensity. Panel (a2) μ_PL_ values raster plots. Each dot represents a single and isolated sperm cell. Red, green and blue, solid and dashed lines correspond to the average of μ_PL_ signals and the standard deviation, respectively. The number of cells considered was n = 130, n = 334 e n = 62 for sample with 45%, 25% and 5% progressive motility, respectively. The grey dashed line represents the noise threshold (fixed as η + 5σ, where η is the noise average and σ its standard deviation). Panel (a3) μ_PL_ intensities distribution plots. Red, green and blue plots are representative of 10 samples for each motility class. The red bold lines represent the average of μ_PL_ signals for sample with 45%, 25% and 5% progressive motility, respectively. Data are normalized according with the % of total number of cells. Panel (b1) Maximum value of photoluminescence intensity on the entire middle piece (M_PL_) intensities from 10 samples for each progressive motility category. The color bar is reported in arbitrary units (A.U.): from blue to red, the bar shows growing M_PL_ values. Therefore, each point of the color map represents the M_PL_ intensity recorded from a single cell in one of the 10 samples. Data are sorted by decreasing cell number and fluorescence intensity. Panel (b2) M_PL_ intensities raster plots. Each dot represents a single and isolated sperm cell. Red, green and blue, solid and dashed lines correspond to the average of M_PL_ signals and the standard deviation, respectively. The number of cells considered was n = 130, n = 334 e n = 62 for sample with 45%, 25% and 5% progressive motility, respectively. The grey dashed line represents the noise threshold (fixed as η + 5σ, where η is the noise average and σ its standard deviation). Panel (b3) M_PL_ intensities distribution plots. Red, green and blue plots are representative of 10 samples for each motility class. The red bold lines represent the average of M_PL_ signals for sample with 45%, 25% and 5% progressive motility, respectively. Data are normalized according with the % of total number of cells.

Single cell μ_PL_ and M_PL_ values for each measured cell in each sample for the three motility groups are reported in Fig. 3a1 and b1, showing how the number of cells with high fluorescence varies in the three different motility classes investigated in this work, allowing also to better compare the two detection methods based on the maximum or the mean fluorescence intensity.

In particular, for the normozoospermic sample M_PL_ data are distributed among much wider intensities set, while for μ_PL_ data are more concentrated across the mean value, leading to a lower standard deviation (see representative cells in Fig. 3a2 for μ_PL_ and Fig. 3b2 M_PL_). Similar considerations are valid also for the asthenozoospermic sample. Instead, in the case of the severe asthenozoospermic sample similar standard deviations were observed for both μ_PL_ and M_PL_ data, with the vast majority of the cells showing a fluorescence well above the noise threshold (this latter was fixed as η + 5σ, where η is the noise average and σ its standard deviation). To better show this point, PL intensity distributions for both analysis methods were calculated for the 10 samples from each progressive motility group, displayed in Fig. 3a3 for μ_PL_ and in figure Fig. 3b3 for M_PL_ with the related average distribution over the 10 samples highlighted in bold. The overall assessment shows a peak left shift according to decreasing progressive motility for both μ_PL_ and M_PL_ with important modifications of the distribution shape, and it confirms a wider distribution of M_PL_ with respect to μ_PL_ for 25% and 45% motility groups.

These results let us suggest that it is not sufficient to refer to the average fluorescence value to assess the quality of the sample, but the fluorescence intensity distribution plays a key role in evaluating the sample quality on the base of fluorescence intensity of MT-G stained mitochondria.

### Effect of molecules acting on MMP

In order to prove that the fluorescence intensity variations we observe through MT-G staining are linked to mitochondrial activity, we treated a sample (25% progressive motility) with two different molecules able to modify MMP: nicotinamide adenine dinucleotide reduced form (NADH) 2 mM and carbonyl cyanide-4-(trifluoromethoxy)phenylhydrazone (FCCP) 0.5 mM to increase or reduce MMP, respectively.

FCCP is an uncoupler of mitochondrial oxidative phosphorylation and reduces MMP, while NADH represents a source of reducing equivalents for the mitochondrial respiratory chain and therefore it hyperpolarizes MMP^25^.

**Figure 4:**
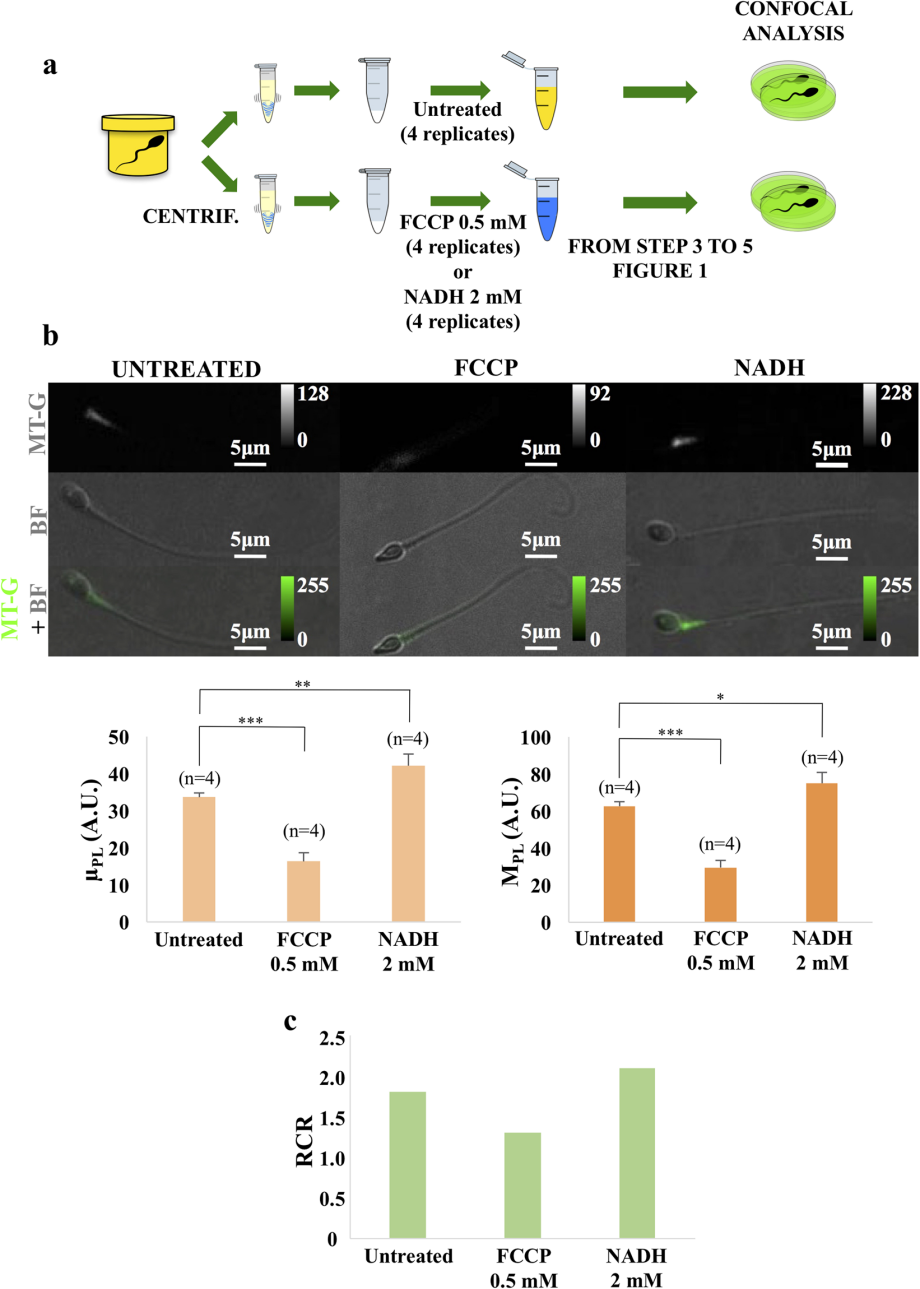
Effects of FCCP and NADH on mitochondrial functionality. Panel (a) Steps of sperm cell samples treatment. From left to right: samples dilution, centrifugation at 10000 g for 5 minutes at room temperature, pellets pre-treatment in presence and in absence of a solution of 0.5 mM of FCCP or 2 mM of NADH, intermediate centrifugation, 200 nm MitoTracker Green FM solution incubation for 15 minutes at 37 °C, a further centrifugation and pellets resuspension in saline water (1 ml). Panel (b) Confocal microscope images of sperm before and after FCCP 0.5 mM or NADH 2 mM incubation. Fluorescence (MT-G), bright field (BF) and overlay (MT-G + BF) channels are displayed. Color scales represent fluorescence intensity in arbitrary units. Bar graphs of average value of photoluminescence intensity on the entire middle piece (μ_PL_) and maximum value of photoluminescence intensity on the entire middle piece (M_PL_) of untreated sperm cells (4 replicates), FCCP 0.5 mM (4 replicates) and NADH 2 mM (4 replicates) treated spermatozoa. Pre-incubation of sperm with FCCP 0.5 mM resulted in a μ_PL_ and M_PL_ intensity significant decrease (***p < 0.001; p = 0.0001 and p = 2.46 × 10^−5^). Pre-incubation with NADH 2 mM resulted in a μ_PL_ and M_PL_ intensity significant increase (**p < 0.01; p = 0.008 and *p < 0.05; p = 0.017). Data are reported as mean value and error bars represent standard deviations on n = 4 replicates. Panel (c) Bar graph of the Respiratory Control Ratio (RCR) in samples before and after FCCP 0.5 mM and NADH 2 mM incubation. In all panels NADH stays for nicotinamide adenine dinucleotide, while FCCP stays for carbonyl cyanide-4-(trifluoromethoxy)phenylhydrazone.

The incubation protocol flows as described in Fig. 4a: one sample (from one donor) was split into 12 tubes, 4 for untreated, 4 for FCCP, and 4 for NADH. In a parallel assay, 15 million of sperm cells were used for the RCR determination. The 12 aliquots were then incubated with MT-G with the protocol described in the previous paragraph.

As shown in Fig. 4b, pre-incubation of sperm cells with FCCP 0.5 mM resulted in a decrease of mean value of μ_PL_ and M_PL_ of about 50% (p < 0.001). On the other hand, when sperm suspensions were pre-incubated with NADH 2 mM a μ_PL_ and M_PL_ increase of about 20% was observed (p < 0.01 and p < 0.05 respectively). Notably, for both NADH and FCCP experiments, the RCR values are coherent with the fluorescence detection: a significant reduction of mitochondria functionality index was found for the sperm cells incubated with FCCP, while an increase of the same parameter was found for the cells for treated with NADH (see bar graphs in Fig. 4c).

## Discussion

We propose a new approach to investigate mitochondrial functionality of sperm cells by combining fluorescent staining of internal mitochondria membrane and laser scanning confocal microscopy (LSC). There are several dyes that could be used to assess mitochondrial membrane potential. We test the ability of MT-G to assess MMP in sperm cells as already suggest by^20,21,26^. Although these outcomes contradict the established indications that MT-G staining is independent of MMP^27^, our results are a further confirmation that the fluorescence intensity of MT-G staining could be linked to MMP, at least in sperm cell, that have a very peculiar mitochondria structure with respect to other cell types^28^.

The resulting PL signal, obtained at the single-cell level from the middle piece and evaluated as the mean value PL intensity over tens of cells in the sample, increases as a function of sample progressive motility. The link between these data and the MMP was confirmed by the RCR analysis, a measure of mitochondrial activity of the sperm cells. These findings match well with previous studies, where approaches based on fluorescence microscopy^20^ or citofluorimetry^11–13,16,18,19^ were used to assess whether a sample has a “high” or “low” level of viability or progressive motility. Our results, instead, suggest that fluorescence investigation of MMP has the potential of allowing for a more detailed analysis, giving access to MMP statistical distribution at the single cell level. One consequence of this is that the mean PL intensity value allows distinguishing between more than two levels of progressive motility, as shown by the bar graphs in Fig. 2b and c for groups with 5%, 25% and 45% of progressive motility. We suggest that this is mainly obtained by virtue of the ability of the LSC to obtain efficient excitation and collection of fluorescence signal from sub-cellular compartments, improving the overall sensitivity and resolution of the detection with respect to previously employed methods. Moreover, this approach allows to access to the statistical distribution of PL intensities among all the investigated cells, as reported in Fig. 3. Our analysis shows an overall heterogeneous population of fluorescence intensity (and therefore of MMP) for all investigated samples, revealing peculiar differences between the investigated normozoospermic, asthenozoospermic and severe asthenozoospermic samples. A screening over several individuals allows us to better assess the statistic distribution of PL among the sample, potentially leading to a method to evaluate sperm quality on a figure of merit directly related to MMP and therefore to energetic efficiency of the single sperm cells.

To confirm that the fluorescence variations we observe are directly related to MMP, we modified the membrane potential by using two difference molecules: FCCP and NADH, with depolarizing and hyperpolarizing effects, respectively. The obtained fluorescence data, shown in Fig. 3, confirm that increasing (reducing) MMP results in a significant increase (decrease) of both PL intensity and RCR values. This further validates the link between MMP and sperm motility found by Paoli *et al*.^22^, who identified a profile of MMP values corresponding to a precise sequence of gradually increasing motility. These findings also agree with previous results by Ferramosca *et al*.^9^, who linked sperm mitochondrial respiration, evaluated by the polarographic assay of oxygen consumption, with variations in sperm motility to establish a profile for RCR values with respect to a gradual increase or decrease in sperm motility. Therefore, the use of confocal laser scanning microscopy to directly evaluate mitochondrial function in sperm samples has also the potential to be exploited for examining molecules of pharmacological interest on sperm quality. In fact, it would be interesting to exploit the here-proposed approach to evaluate the effects of different molecules and drugs in order to test beneficial effects in the treatment of male infertility or any dangerous effect on sperm quality.

In summary, we present a method to identify mitochondria functionality at the single-cell level and to link different degrees of progressive motility to different levels of mean PL intensity from sperm-cell mitochondria. This approach allows to access the statistics of PL intensity over hundreds of cells and to draw conclusions on sample quality also based on PL statistical distribution among the sample. Since MT-G intensity is consistent between different days of experiment, this assay could be used routinely for assessing sperm quality.

## Materials and Methods

### Human semen samples

The use of semen was allowed by the donors with written informed consent. This research was approved by the Department of biological and environmental sciences and technologies at the University of Salento. All experiments were performed in accordance with the relevant guidelines and regulations for research on human subjects.

For this study, sperm cells samples and respective spermiograms were provided by “Pignatelli” laboratory of clinical analysis in Lecce and by biological medical center “Tecnomed” in Nardò (Lecce), Italy. Semen samples were collected after 3–5 days of sexual abstinence, by masturbation and examined directly after liquefaction in a period <30 minutes. Semen analysis was performed according to World Health Organization guidelines^3^. Automated computer analysis of sperm motility (CASA - SCA: Sperm Class Analyzer, LabIVF Asia Pte Ltd, Singapore) was carried out on all semen samples.

For this study, we selected the sperm by evaluating only the progressive motility (with an instrumental error of +/− 0.5%), excluding cases with particular pathologies.

### Treatment of sperms with molecules acting on MMP

Fresh sperm suspensions were centrifuged (10.000 g for 5 minutes, at 37 °C) to obtain a cell pellet and the supernatant was subsequently aspirated.

Sperm cells were separately incubated in either the presence or absence (controls) of 0.5 mM carbonyl cyanide-4-(trifluoromethoxy) phenylhydrazone (FCCP) and 2 mM nicotinamide adenine dinucleotide reduced form (NADH) for 15 minutes at 37 °C.

FCCP stock solution was made in ethanol, control experiments for this chemical included similar amounts of ethanol.

### Staining with MitoTracker Green FM and LSC analysis

Samples were centrifuged (10.000 g for 5 minutes, at 37 °C) to obtain a cells pellet and the supernatant was subsequently eliminated. The pellets were resuspended in prewarmed (37 °C) saline water containing 200 mM of the probe MitoTracker Green FM and incubated for 15 minutes under growth conditions. Samples were centrifuged again (10.000 g for 5 minutes, at 37 °C) and cells resuspended in fresh prewarmed saline water (Fig. 1a). This method was reproduced following the protocol already described in ref.^20^, an approach that preserves sperm cells viability.

The sample was then cast in a Fluoro Dish (50 mm diameter; WPI) previously treated with a Plasma Cleaner (Diener – Plasma Surface Technology) to improve inner surface hydrophilicity.

Samples were analyzed by a confocal laser microscope (LEICA TCS SP8 X). Images were acquired with the LasX Software using a 100X oil-immersion objective. The pinhole was set at 1 Airy unit (95 μm). A 490 nm continuous wave diode laser was used for sample excitation with a power of 12%. Fluorescent emission was detected in the spectral window between 500 nm and 550 nm by a GaAsP photomultiplier tube (PMT). 116.25 × 116.25 μm wide images were acquired at 1024 × 1024 pixels with a pixel size of 113.64 × 113.64 nm, scan speed of 200 Hz. Z-stacks were acquired with a z-step size of 0.5 μm for a total z-lenght of 2 μm. The average projection of all images, for each field of view, was performed with the open source software ImageJ.

### Mitochondria respiration studies

Spermatozoa were collected by centrifugation at 800 g for 10 minutes at room temperature and washed by resuspension in isotonic salt medium (2 g/L bovine serum albumin, 113 mM KCl, 12.5 mM KH_2_PO_4_, 2.5 mM KH_2_PO_4_, 3 mM MgCl_2_, 0.4 mM ethylenediaminetetraacetic acid, and 20 mM tris adjusted to pH 7.4 with HCl). They were then subjected to hypotonic treatment essentially as previously described.

Oxygen uptake by hypotonically treated spermatozoa was measured at 36 °C by using a Clark-type oxygen probe (Oxygraph, Hansatech Instruments, King’s Lynn, UK), in the presence of mitochondrial respiratory substrates (10 mM pyruvate and 10 mM malate) and 0.76 mM of adenosine diphosphate (ADP). The rate of oxygen uptake by sperm mitochondria (V) was expressed as nmol O_2_ mL^−1^ × minute^−1^. The respiratory control ratio (RCR) was calculated by dividing V_3_ (rate of oxygen uptake measured in the presence of respiratory substrates + ADP) by V_4_ (rate of oxygen uptake measured with respiratory substrates alone).

### Statistical analysis

In this study, we used 10 sperm samples for each progressive motility group (5%, 25% and 45%). All the data are reported as mean value ± the standard deviation. We calculated the p-values through the EXCEL function Student’s t-test (T.TEST) to evaluate if the fluorescence signals detected from each group are significantly different from each other. We considered the following significance levels: ns, p > 0.05, *p < 0.05, **p < 0.01, ***p < 0.001.
